# Mathematical Modelling of Tensile Mechanical Behavior of a Bio-Composite Based on Polybutylene-Succinate and Brewer Spent Grains

**DOI:** 10.3390/polym16212966

**Published:** 2024-10-23

**Authors:** Annamaria Visco, Cristina Scolaro, Francesco Oliveri, Aldo Jesus Ruta

**Affiliations:** 1 Department of Engineering, University of Messina, C.da Di Dio, 98166 Messina, Italy; 2 Institute for Polymers, Composites and Biomaterials—CNR IPCB, Via Paolo Gaifami 18, 9, 95126 Catania, Italy; 3 Department of Mathematical and Computer Sciences, Physical Sciences and Earth Sciences, University of Messina, Viale F. Stagno d’Alcontres, 98166 Messina, Italy; francesco.oliveri@unime.it (F.O.); aldojesus.ruta@unime.it (A.J.R.)

**Keywords:** bioplastics, poli-butylene-succinate, agri-food waste, tensile test, mathematical modelling

## Abstract

A model based on the fitting of stress–strain data by tensile tests of bio-composites made of a bioplastic (polybutylene succinate (PBS)) and brewer spent grain filler (BSGF) is developed. Experimental tests were performed for various concentrations of BSGF in the range from 2% to 30%. The model is suitable for describing the elastic–plastic behavior of these materials in terms of two mechanical parameters, tensile stress and tensile stiffness (or Young’s modulus), depending on the filler concentration. The mechanical characteristics, derived from the fit parameters, show good agreement with the experimental data. The mathematical model used here could be an important aid for the experimentation and manufacturing process as it allows the prediction of the mechanical tensile parameters of a mixture with different filler concentrations, avoiding the long and complex preparation cycle of bio-composites, as well as the specific mechanical tests. The physical properties required by the objects created with the PBS–BSGF bio-composite by the partners/stakeholders of the research project co-financing this research can be quite different; therefore, a mathematical model that predicts some of the mechanical properties in terms of the mixture composition may be useful to speed up the selection of the required amount of BSGF in the mixture.

## 1. Introduction

It is well-known that Fossil Based Plastics (FBP) exhibit many positive features: low cost, easy availability, easy processing of raw materials, and good mechanical properties. These aspects have resulted in a real explosion in the use of FBPs beginning in the 1950s, replacing traditional materials such as glass or metals that presented problems of fragility, heaviness, or corrosion [1]. Unfortunately, FBPs are non-biodegradable materials and have a very long lifespan (hundreds or thousands of years) [2].

Current data [3] indicate that FBP production will be on the order of about 500 million tons in 2025. The lack of recycling and poor management of plastic waste due to the linear economy model [4] of the past decades have led to well-known environmental pollution problems throughout the world. Furthermore, excessive industrial production is one of the main causes of global warming, with climate consequences that we are already experiencing [5]. The huge amount of plastic waste has affected the ecosystem due to pollution in soils and oceans (through landfills) and in the atmosphere (through fumes from industrial production) [6].

To overcome these serious problems, it has become necessary to increase the use of alternative resources with respect to fossil ones. The biological origin of bioplastics represents an alternative to fossil-based plastics for their biodegradability [7]. Biodegradable and Bio-Based Polymers (BBPs) from renewable resources are an interesting alternative to traditional FBPs, due to their much faster biodegradation in the soil compared to FBPs [8,9,10]. This feature is crucial for eliminating the serious problem of FBP pollution and, in this way, contributing to the protection of the ecosystem and the health of the entire planet [10]. Although the production cost of bioplastics is progressively decreasing, it is still higher than that of plastics derived from fossil fuels [7].

In any case, to satisfy the production of millions of tons of bioplastics, it would be necessary to shift a large part of the production of natural resources, which could no longer be used for the food needs of the world population [11,12,13].

Bio-polybutylene succinate (PBS), obtained from renewable sources, is a white, ductile, semi-crystalline material that behaves like a thermoplastic (i.e., like low-density polyethylene (LDPE)) and is also biodegradable. Thus, it represents a valid alternative to FBPs because of its physical/mechanical properties [14]. Moreover, low production costs and the possibility of obtaining both succinic acid and 1,4-butanediol from renewable sources makes PBS a completely biobased polymer [1]. In detail, PBS has a density of 1.25 g/cm^3^, a melting point (Tm) in the range of 90 °C to 120 °C, and a glass transition temperature (Tg) in the range of −45 °C to −10 °C [15].

For the issues above discussed (a reduction of the use of virgin polymers, an increase of biodegradability, and a reduction of production costs), a promising strategy is to mix polymeric materials with Ligno-Cellulosic Fillers (LCF) such as wheat bran, jute, bamboo, rice straw, or hemp. Because these fillers are agri-food production waste, they are practically zero-cost materials [16,17,18,19,20].

The presence of an inert filler in the bio-composite has two main consequences: on the one hand it reduces the consumption of bioplastic, on the other hand it is responsible for changing both the mechanical and physical properties of the thermoplastic material [21].

The bio-composite does not contain any chemical additive (potentially dangerous or toxic) to lower the interfacial tension between the PBS (which is non-polar) and the BSGF (which is polar).

To describe the mechanical behavior of the PBS mixture with LCF, we performed a series of experimental tests accompanied by a modeling analysis. The mechanical parameters (temperature, pressure, and processing parameters being constant) are strictly linked to the composition of the polymer/filler mixture and, therefore, depend both on the degree of dispersion of the filler within the polymeric material and the chemical affinity between the two components of the mixture [22,23].

To avoid both the lengthy experimental preparations of all possible combinations of the PBS/LCF mixtures and, therefore, limiting to a minimum the experimental characterization test, we analyzed the stress–strain data obtained for a small (although significant) number of different compositions of the PBS/LCF mixtures by using the same fit function independently of the filler concentration. To obtain our results, we considered fifteen different concentrations of the filler (from 2% to 30%) and performed experimental mechanical tests by using four samples for each composite. The fit function we explored in the data analysis can be useful for describing the general mechanical behavior of the PBS/LCF composite and, hopefully, give good predictions for compositions of mixtures different from those used in the experiments.

In the literature, there are many contributions related to the mathematical modeling of composite materials. Fundamental results about elastomers [24,25,26,27,28] are worth quoting: these materials were, and still are, relevant, especially in the automotive and transport industry, but also in the mechanical, textile, and chemical industries. In even more detail, in-depth mathematical modeling studies were carried out to analyze the macromolecular structure of polymer chains [29,30,31]. Viscoelastic solids can be described theoretically using well-known models that put together a purely viscous damper and a purely elastic spring: these two basic elements are connected in series in the Maxwell model [32], or in parallel in the Kelvin–Voigt model [33]. More sophisticated models are also possible [34]. Work is in progress to use a general theoretical model to account for experimental data.

A complete analysis of the literature is impossible. Hereafter, we quote a number of papers that share certain features with our work. Alasfar et al. [35] developed a mathematical model of porous polymer nanocomposites, which examines how mechanical properties can be influenced by temperature, material porosity, operating conditions, and loading rate. Potluri et al. [36] used finite element methods (FEM) for the prediction of the elastic properties of a pineapple fiber-reinforced polymer composite under unidirectional load.

Kazmer et al. [37] modelled the process property relations of different commercially available bioplastics (a recycled polypropylene blend, polybutylene adipate terephthalate, and two grades of polylactic acid) in a pilot production environment using an instrumented two-cavity hot runner injection mold. Zhou et al. [38] investigated the effect of different waste Glass Fiber-Reinforced Polymer (GFRP) volume replacement ratios on the performance of concrete. They studied, in detail, the failure mode, uniaxial compressive strength, modulus of elasticity, lateral dilation, and stress–strain response.

It is easy to recognize that a mathematical description of the mechanical behavior of composites that can aid in the development of new materials—such as those based on PBS and a lignin cellulosic filler, like the brewer spent grain filler (BSGF) that represents an agri-food waste reusable for other purposes—is very important. In this paper, we try to address some issues concerned with a simple mathematical description of the mechanical behavior of the PBS–BSGF mixtures.

The innovative feature of this paper, mainly concerned with the mathematical analysis of the experimental results, consists in the unique fit function for describing the stress–strain data in compounds with different concentrations of the filler. In fact, for filler weight concentrations ranging from 2% to 30%, the same functions, containing only two parameters that appear to be linearly related to the concentration of spent beer grains, can fit the experimentally obtained stress–strain curves. The results presented in this paper, and in a forthcoming paper, will serve to construct a constitutive theory of such compounds within the framework of continuum mechanics [39].

## 2. Materials and Methods

### 2.1. Materials and Morphological Investigation

Bioplastic: Bio-PBS FZ71 (code: PBS) was purchased from Mitsubishi Chemical Performance, FZ71PM grade, with a density of 1.260 g/cm^3^ and melt flow rate (MFR) of 22 g/10 min (190 °C/2.16 kg).

Filler: brewer spent grain (code: BSGF) was supplied by Birrificio Messina Società Cooperativa (Larderia Inferiore, ex ASI, n.4, 98129 Messina, Italy) and was ground by ball milling (RETSCH mod. MM301, operative conditions: 20 Hz, 30 min). Then, the BSGF was sieved with a Filtra Vibration apparatus, mod. Filtra IRIS FTL-0200 until a grain size smaller than 100 µm was obtained.

Visual examination of BSGF powder and of the fracture surface of dog bone shaped specimens used for tensile testing (described in Section 2.3) was performed by a Scanning Electron Microscope (SEM). A ZEISS Crossbeam 540 microscope (Carl Zeiss Microscopy GmbH, 07745 Jena, Germany) was used for this analysis. The operating conditions were as follows: accelerating voltage of 5 kV, magnifications of 300×, 3000×, and 8000×, and coating of the specimens with a thin layer of chromium (Quorum Q 150T-ES, Quorum Technologies Limited Company, West Sussex RH19 2HL, UK).

**Figure 1:**
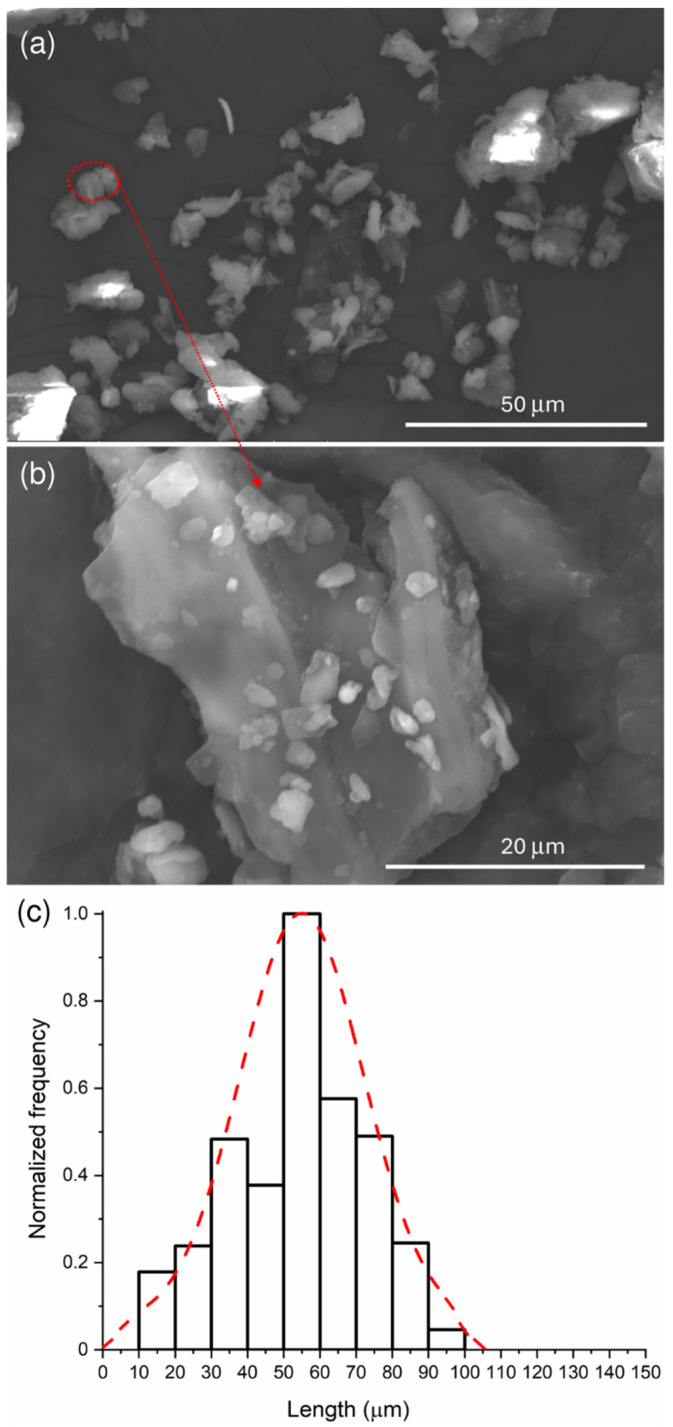
SEM analysis at 3000× (**a**), at 8000× (**b**), and size distribution of BSGF (**c**).

SEM micrographs of BSGF powder at two different magnifications (3000× and 8000×) are shown in Figure 1a,b, respectively. BSGF powder has an irregular shape that extends along one main direction and is therefore preferentially elongated. The enlargement of Figure 1b, in fact, shows a rectangular-shaped particle, approximately 38 μm long and about 26 μm wide, which is made up of a block covered in turn by agglomerates of even smaller dimensions (a few micrometers) deposited on its surface.

The average distribution of the length of the BSGF powder particles was calculated on 569 particles by using ImageJ software (version 1.53 k-java8, National Institute of Mental Health, 9000 Rockville Pike, Bethesda, MD, USA). The Gaussian distribution (shown in Figure 1c) was obtained by OriginPro2024 software (OriginLab Corporation, One Roundhouse Plaza, Suite 303 Northampton, MA, USA). The maximum length value of the dimensional distribution curve is at 54 ± 18 μm (Figure 1c). The particle length size ranges from about 6 μm to about 99 μm.

### 2.2. Bio-Composite Preparation

**Figure 2:**
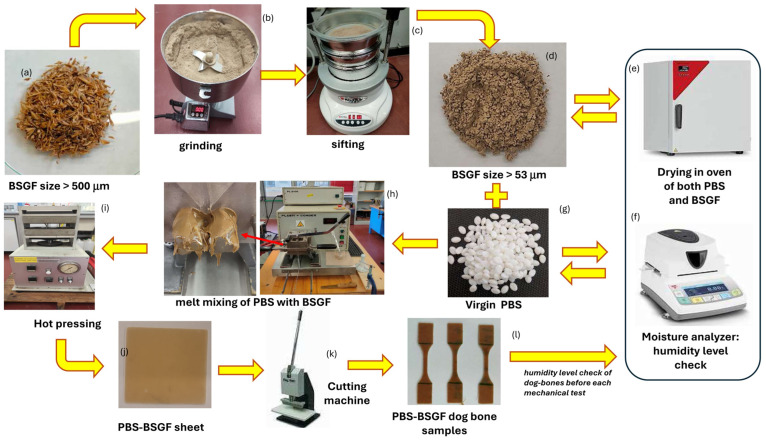
Scheme of production of PBS–BSGF, from raw materials to bio-compound: the processing of BSG to obtain a fine power (size: 53 micrometer) (**a**–**d**), the drying and checking of the humidity level (**e**,**f**), the melt-mixing of BSGF with PBS (**g**,**h**), the production of sheet (**i**,**j**) and of dog-bone (**k**,**l**).

Figure 2 shows the entire long and complex process of preparing bio-composites, from the treatment of the raw materials, to the treatment and control of the humidity level, to the preparation of the mixture and the shaping of the samples.

Due to the high hygroscopicity of the raw materials, especially of the BSGF, both PBS and BSGF were pre-dried (BSG overnight at 80 °C and PBS 4 h at 60 °C) in an oven (Binder Avantgarde line, mod. ED56) before processing, and the humidity level was checked with a moisture analyzer (ATS120) to keep the humidity level below 2 wt%. A higher level of humidity can negatively affect the creation of the product during technological processing operations.

The BSGF must be stored in a vacuum bag to prevent it from absorbing further humidity. Moreover, before each processing, it is necessary to recheck the humidity level as it easily absorbs humidity, similarly to a sponge.

The blend of PBS–BSG was prepared from a direct melting process in a melt-mixer machine schematized in Figure 1: PBS, both pure and with BSGF at different weight amounts ranging between 2 wt.% and 30 wt.%, were mixed in a Brabender Plasticorder PL2100 chamber at 140 °C, speed 40 rpm, for ten minutes.

**Figure 3:**
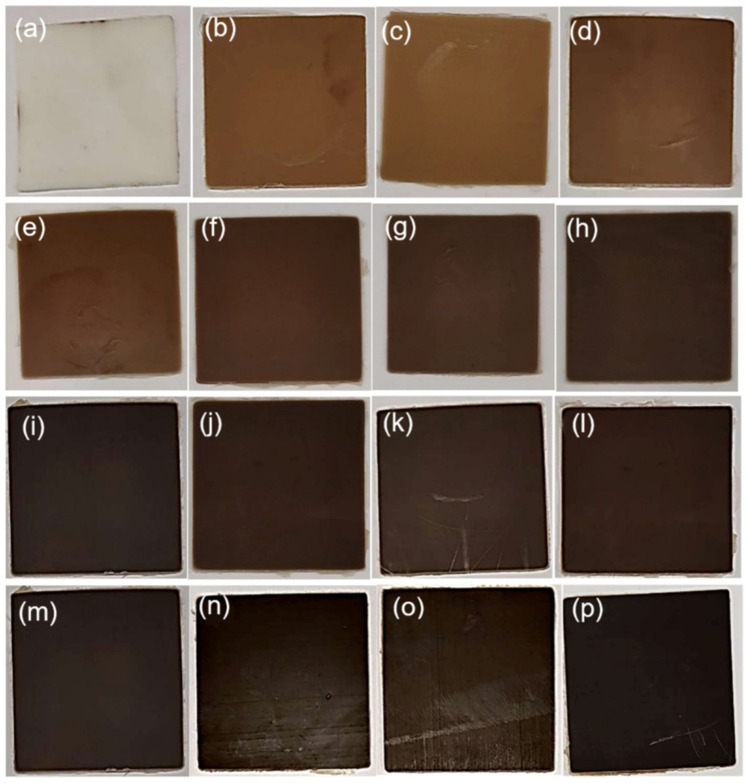
Sheets of PBS (**a**), PBS–BSGF2 (**b**), PBS–BSGF4 (**c**), PBS–BSGF6 (**d**), PBS–BSGF8 (**e**), PBS–BSGF10 (**f**), PBS–BSGF12 (**g**), PBS–BSGF14 (**h**), PBS–BSGF16 (**i**), PBS–BSGF18 (**j**), PBS–BSGF20 (**k**), PBS–BSGF22 (**l**), PBS–BSGF24 (**m**), PBS–BSGF26 (**n**), PBS–BSGF28 (**o**), PBS–BSGF30 (**p**).

**Figure 4:**
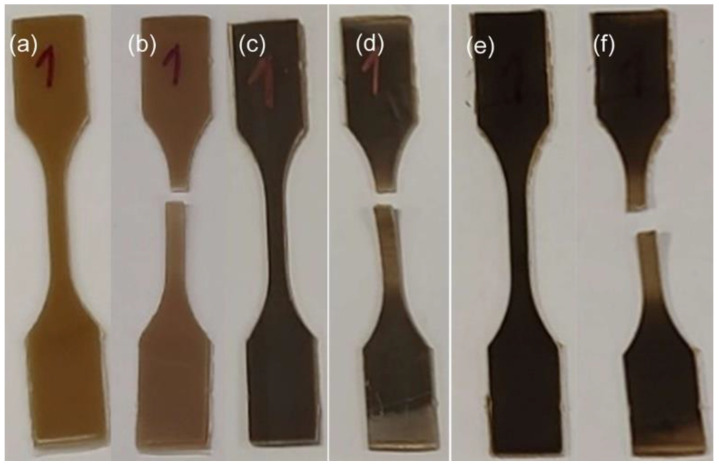
Dog-bones blends of PBS–BSGF4 (**a**,**b**), PBS–BSGF20 (**c**,**d**), PBS–BSGF30 (**e**,**f**) before and after the tensile test.

The resulting blends (Figure 2) were thermoformed in a uniaxial hot press (PM 20-200, supplied by DGTS s.r.l. Veduggio Con Colzano (MB) Italy) at 140 °C for 15 min, at different pressures, with the following sequence: 7 min at atmospheric pressure (just putting the mold in contact with the heating plates), 5 min at 50 bar, and 3 min at 100 bar to obtain 6 × 6 cm square sheets, 1 mm thickness (Figure 3). Dog-bone samples were obtained by a Ray-Ran cutter machine according to international standard ASTM D638-03 [40] (Figure 4).

### 2.3. Mechanical Characterization Tests

Tensile tests were performed according to the ASTM D638-03 standard with a Lloyd LR10K Universal Dynamometer machine (load cell 0.5 kN, preload 1.00 N, speed 2 mm/min) purchased from Elis–Electronic Instruments & Systems S.r.l., Rome, Italy.

Tests were carried out at 25 °C and a relative humidity (RH) of 21%. Mechanical parameters such as Young’s modulus (E (MPa)) and stress at break (σ_r_ (MPa)) were obtained as the result of the average values obtained from four samples (for each type).

### 2.4. Data Analysis

**Figure 5:**
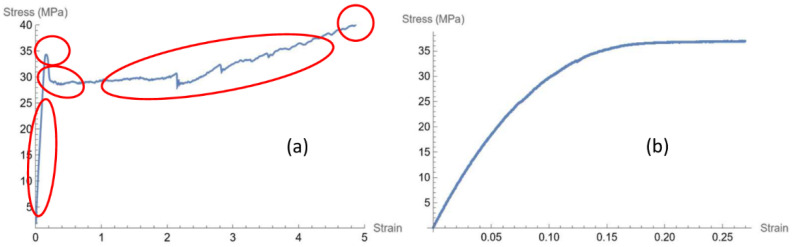
Engineering stress–strain curves of PBS (**a**) and PBS–BSGF4 dog-bone specimens (**b**).

Looking at the engineering stress–strain curve of pure PBS, one can recognize different phases: proportional limit, yield point, necking, strain hardening, and rupture, underlined in red in Figure 5a, respectively.

On the contrary, when the filler is mixed with the polymer, even at low concentration, the outcoming composite shows a totally different mechanical behavior, as can be observed from the stress–strain curve in Figure 5b. In fact, the stress–strain curves, for all concentrations of the filler used, show that a plateau for the stress is reached until failure. At present, we did not experimentally check the value of the stress where a plastic deformation occurs since all the bio-composites do not show a yielding point.

In the literature, a well-known model which describes the stress–strain relationship of elasto-plastic materials is based on the Ramberg–Osgood relationship [41], which is a phenomenological model requiring a check with experimental data.

In a more recent work [42], within the framework of thermo-mechanics with internal variables, a mathematical function is used to fit the stress–strain curve of paper (bi-axial tensile test) and implement a constitutive model.

Based on these papers, our starting idea was to fit the data of the stress–strain curves with a nonlinear fit function, and then collect the fit parameters in order to find a relation between the fit parameters and the concentration of the filler. In fact, when dealing with a mixture, it is convenient to describe each mixture by using the concentration of the filler expressed as a fraction (mass of filler/total mass).

As one expects, the stress–strain curves of these blends present nonlinear behavior.

For each batch of samples at the different filler concentrations given in Table 1, we analyzed stress–strain relationship obtained from experiments and determined the material parameters α and β involved in the fit function. The fitting procedure is carried out with the data provided by the tensile test machine of four dog bones for each filler concentration considered, using the function (see [36])

(1) $$\sigma =\alpha \;tanh\left(\beta \varepsilon \right)$$

as the model of fit, where σ denotes the stress (MPa), ε the strain, and α and β are constants to be determined by using a nonlinear least squares method using the Levenberg–Marquardt algorithm [43] (actually, the results were obtained by means of “FindFit” function of Wolfram Mathematica [44]).

From relation (1), one can compute Young’s modulus by means of Formula (2) and compare it with the corresponding experimental value.

(2) $$E=\frac{d\sigma }{d\varepsilon }{|}_{\varepsilon =0}=\alpha \;\beta$$

The latter equation represents the derivative of Equation (1) with respect to the strain variable evaluated at strain equal to zero.

In the next Section, the analysis of the experimental stress–strain data for the various tested concentrations of the filler is discussed.

## 3. Results and Discussion

In this Section, as a result of fitting the stress–strain data for all the considered samples with the same family of functions, we realize that the numerical values of the parameters depend on the concentration of the filler, i.e., at least in the range of 2–30% in the experimental setting (tensile test at a specific strain rate of 2 mm/min). In particular, the parameter α has a decreasing trend for increasing values of the concentration of the filler, whereas β has an increasing trend.

**Figure 6:**
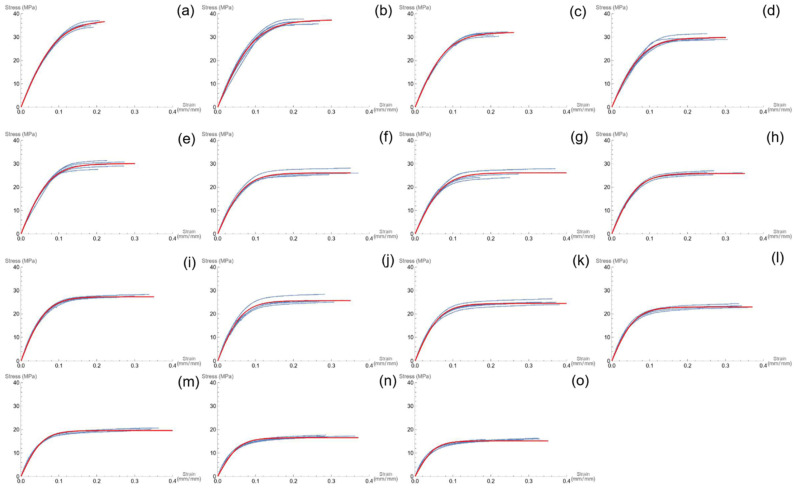
Stress–strain curves (blue) and non-linear were curve (red) of bio-compounds at different concentration of BSGF filler, from left to right: PBS–BSGF2 (**a**), PBS–BSGF4 (**b**), PBS–BSGF6 (**c**), PBS–BSGF8 (**d**), PBS–BSGF10 (**e**), PBS–BSGF12 (**f**), PBS–BSGF14 (**g**), PBS–BSGF16 (**h**), PBS–BSGF-18 (**i**), PBS–BSGF20 (**j**), PBS–BSGF22 (**k**), PBS–BSGF24 (**l**), PBS–BSGF26 (**m**), PBS–BSGF 28 (**n**), PBS–BSGF30 (**o**).

Figure 6 displays the experimental stress–strain curves of all the bio-composite materials. The curves show that the slope of the linear part (Young’s modulus) of the curve progressively decreases for increasing concentration of the filler, and the same trend applies also to the maximum stress.

The numerical values of the parameter (α,β), the experimental mechanical parameter (stress at break and Young’s modulus), and Young’s modulus, computed according to (2), are reported in Table 2.

The plot of *α* vs. BSG content (expressed as the fraction c) shows a linear dependence. In fact, a least square linear regression gives the following relation:

(3) α=−69.06 c+37.50

ρ=−0.955 being the Pearson Correlation Coefficient (PCC).

**Figure 7:**
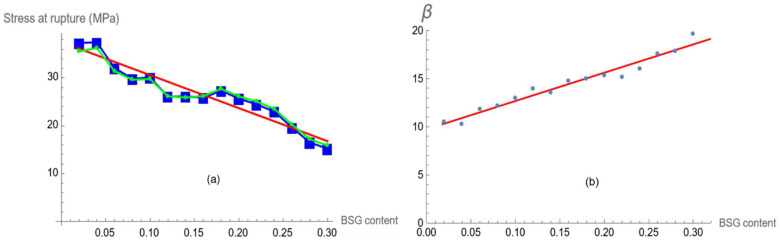
*α*, stress at rupture vs. BSG concentration: experimental points (green line–spot), *α* parameter (blue line–square dot), linear regression function (red continuous line) (**a**). *β* parameters (blue spots) and the linear fitting (red continuous line) (**b**).

Figure 7a displays the plot of the experimental value of Stress at rupture, α, and its linear regression vs. filler concentration (3).

The plot of *β* vs. BSG content shows a linear dependence too. In fact, a least squares linear regression provides the following relation:

(4) β=29.45 c+9.73

ρ=0.979 being the PCC.

The correlation coefficients for the linear dependence of α and β on the filler concentration c are very good. The parameter α mathematically represents the steady state of the fitting curve, represented by (1). Since the stress–strain curve of the compound is almost accurately described by the latter equation, one can assume that α represents the stress at rupture of the material, since the hyperbolic tangent for positive values of its argument rapidly approaches the value 1; this is also confirmed by the optimal match between α and the experimental value shown in Figure 7a.

**Figure 8:**
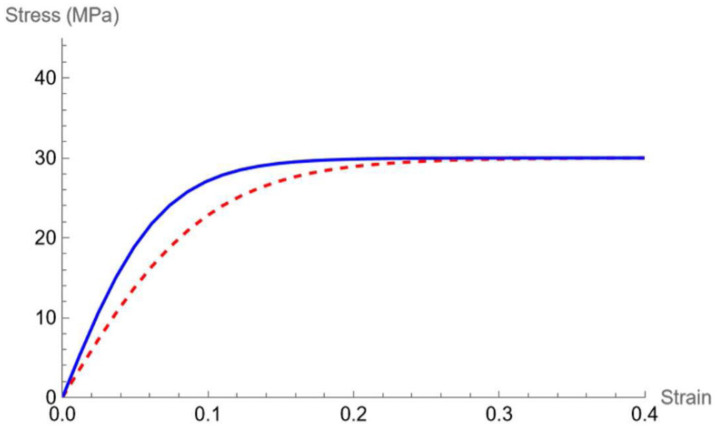
*α* = 30, *β* = 10 (dashed red line), *α* = 30, *β* = 15 (continuous blue line).

The trend of β as a function of filler content is also linear (see Figure 7b) and is represented by Equation (3). The mathematical meaning of this parameter acts as a growth factor, i.e., how fast the dependent variable (stress) reaches saturation: the greater the value of β, the sooner the maximum value of stress is attained, which means the maximum stress is reached at a lower strain. This feature is displayed in Figure 8.

Young’s modulus according to Equation (2) is directly related to the fit parameters (α and β) which are described by means of Equations (3) and (4). As a matter of fact, the product of these two functions gives a second order polynomial:

(5) $$E=-2033.68c^{2}+432.63c+364.79\;\;\;\;\;\;$$

with $R^{2}=0.621$ and ${\mathrm{adjusted}\;R}^{2}=0.592$

**Figure 9:**
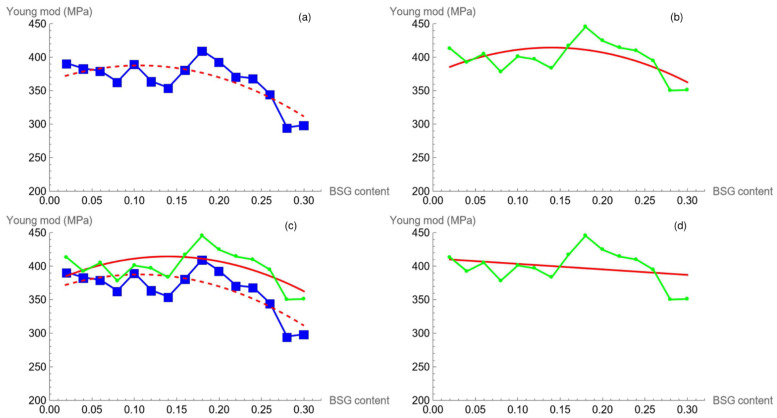
Model (square blue dot) and the polynomial in Equation (5) (dashed red line) vs. BSG content (**a**); experimental (green line–spot) and its nonlinear fitting according to (6) (continuous red line) vs. BSG content (**b**); superposition of the plots shown in Figure 9a,b (**c**); experimental (green line–spot) and its linear regression according to (7) (continuous red line) vs. BSG content (**d**).

The previous equation is plotted against the filler content (Figure 9a) to show the differences between the points (each point represents the product between α and β at the corresponding BSG content) and the function used.

To have a comparison between the experimental Young’s modulus (taken as the linear fitting on the initial part of the stress–strain curve) and the modelling procedure, we fit the experimental points with a polynomial of the same order, resulting in (6), which gives the following equation:

(6) $$E=-2015.33c^{2}+563.18c+375.17\;\;\;\;\;\;\;$$

with $R^{2}=0.376$ and ${\mathrm{adjusted}\;R}^{2}=0.328$

The result, shown in Figure 9b, is qualitatively the same. Instead, a global comparison which considers experimental data, *α*, *β*, and Equations (5) and (6) (Figure 9c) puts in evidence the good agreement between the model and the experimental data.

The nonlinear trend of stiffness (which is instead described by a polynomial) can be related to the poor homogeneity of dispersion of the filler within the bio-composite mixture. This is because PBS and BSGF are not chemically compatible, as already discussed in Section 1. In fact, PBS is a semi-crystalline apolar molecule while BSGF (mainly composed of cellulose and hemicellulose) is amorphous and polar [45,46]. New experimental stress–strain data, obtained using a different chemical formulation of the mixture, show that the Young’s modulus can be estimated by a linear decreasing function of the filler concentration. This will be the object of a forthcoming paper still in preparation.

**Figure 10:**
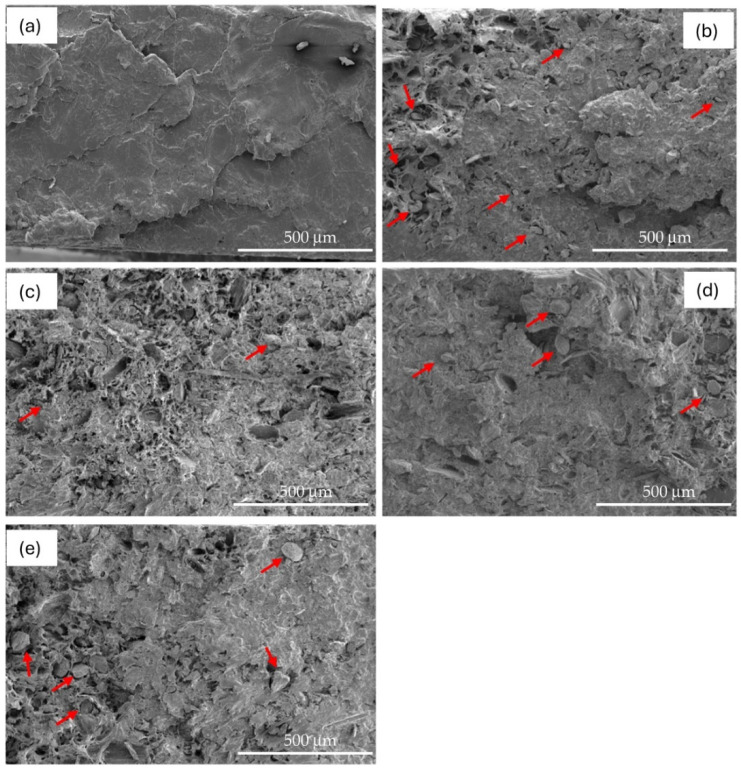
SEM micrographs at 300× of: PBS (**a**), PBS–BSGF10 (**b**), PBS–BSGF20 (**c**), PBS–BSGF25 (**d**), PBS–BSGF30 (**e**).

The lack of homogeneous distribution of BSGF within the PBS matrix in the PBS–BSGF10-20-25-30 bio-composites compared to the pure PBS reference sample can be visualized through the SEM investigation in Figure 10.

If a least squares method is used for fitting the experimental Young’s modulus in terms of the filler concentration c, we obtain the relation

(7) E=−81.73 c+411.71       

ρ=0.286 being the PCC.

When BSGF is inserted into the PBS matrix, the smooth surface typical of the bioplastic (Figure 10a) begins to be modified due to the presence of BSGF and its detachment after the fracture (Figure 10b).

As the content of BSGF increases, the progressive increase of perforated areas in the fracture surface can be easily visualized (Figure 10c–e). In any case, the distribution of voids or particles attached to the matrix is not uniform, regardless of BSGF amount.

However, some BSGF particles appear to be incorporated into the PBS (see the red arrows in the images). Therefore, the BSGF and the PBS appear not to be totally unrelated to each other.

**Figure 11:**
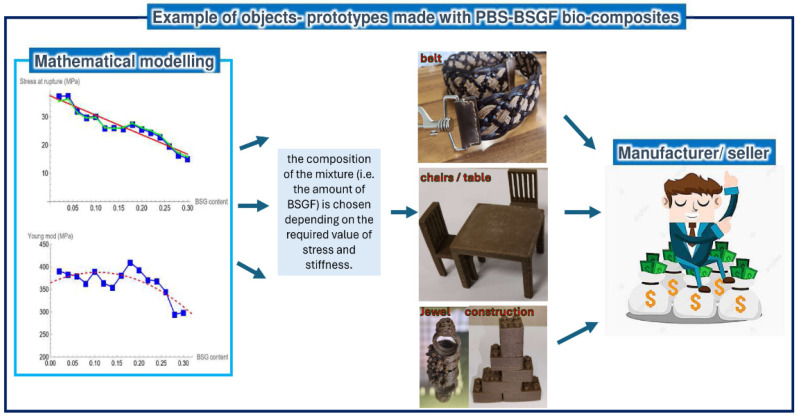
How the mathematical model can help the object production with some examples of prototypes made by some partners/stakeholder of the Life Restart Project with the PBS–BSGF material of this study [47].

Finally, despite the poor compatibility between BSGF and PBS, several prototypes of objects were realized with the PBS–BSGF mixtures investigated in this paper (see Figure 11). The mathematical modeling helps to define the optimal quantity of filler to obtain the desired mechanical performance in terms of mechanical resistance and stiffness. Of course, there are some obvious limitations. The function of fit is not applicable to pure PBS (c = 0); moreover, the validity of the mathematical model is restricted to interpreting the experimental data obtained as described in the paper (e.g., fixed strain rate equal to 2 mm/min) and for filler concentrations in the range of 0.02 to 0.3. In addition, this model cannot evaluate the elongation at break because the fit function (Equation (2)) is made at a constant elongation value that is 0.4 mm/mm.

## 4. Conclusions

The problems related to both environmental pollution caused by non-biodegradable fossil-based plastics and the necessity of disposing of huge quantities of agri-food waste produced daily by the agri-food chain are well known. Based on the above premises, mixtures based on bioplastics (bio-PBS) and agri-food waste (for instance, beer spent grain filler, BSGF) were prepared.

The focus of this work was the study of the mechanical tensile behavior of the mixtures with variable amounts of BSGF (from 2 to 30 percent weight) with the aim of deriving mathematical relations that could model the mechanical behavior of these mixtures.

Despite the lack of optimal dispersion of the lignin–cellulose filler inside the polymer matrix, the mechanical resistance of mixtures was found to be effective in producing bio-composite. Remarkably, it was possible to propose a mathematical model to describe the mechanical behavior of these materials in terms of two parameters that depended on the concentration of the filler.

The mathematical framework we used may effectively be useful in the production process as it allows the prediction of the mechanical traction parameters of a mixture with different concentrations of the filler, avoiding long and complex preparations and specific mechanical tests.

Further investigations on similar mixtures, in which the chemical formulation is modified to make the distribution of the filler in the matrix more homogeneous, are in their final stages. The mathematical analysis of these experimental results will be the subject of a forthcoming paper which is in preparation.

## Figures and Tables

**Table 1:** Composition of all the blends tested in this paper.

Sample		Composition	
**N.**	Code	PBS (wt.%)	BSGF (wt.%)
1 2	PBS PBS–BSGF2	100 98	0 2
3	PBS–BSGF4	96	4
4	PBS–BSGF6	94	6
5	PBS–BSGF8	92	8
6	PBS–BSGF10	90	10
7	PBS–BSGF12	88	12
8	PBS–BSGF14	86	14
9	PBS–BSGF16	84	16
10	PBS–BSGF18	82	18
11	PBS–BSGF20	80	20
12	PBS–BSGF22	78	22
13	PBS–BSGF24	76	24
14	PBS–BSGF26	74	26
15	PBS–BSGF28	72	28
16	PBS–BSGF30	70	30

**Table 2:** Fit parameters (α,β), stress at break from experimental data (σr_exp_)**,** Young’s modulus from the model (E_model_) and from experimental data (E_exp_).

% Filler	α (Conf. Interval) (MPa)	σr_exp_ ± Std Dev (MPa)	β (Conf. Interval)	E_model_ (MPa)	E_exp_ ± Std Dev (MPa)
2%	37.35 (37.29, 37.40)	35.54 ± 1.05	10.47 (10.43, 10.51)	391.17	413.48 ± 11.38
4%	37.43 (37.38–37.49)	36.28 ± 1.00	10.24 (10.20–10.29)	383.40	392.63 ± 32.80
6%	32.09 (32.05–32.12)	31.41 ± 0.75	11.83 (11.80–11.87)	379.73	405.20 ± 11.02
8%	29.82 (29.78–29.86)	29.64 ± 0.99	12.19 (12.14–12.24)	363.50	378.52 ± 23.61
10%	30.13 (30.08–30.18)	29.77 ± 1.49	12.95 (12.89–13.02)	390.26	401.13 ± 27.58
12%	26.11 (26.07–26.16)	26.16 ± 1.46	13.95 (13.86–14.05)	364.33	396.99 ± 20.88
14%	26.17 (26.11–26.22)	25.91 ± 1.84	13.55 (13.46–13.65)	354.66	383.51 ± 11.60
16%	25.85 (25.83–25.88)	26.20 ± 0.63	14.76 (14.71–14.81)	381.69	417.00 ± 14.68
18%	27.36 (27.34–27.38)	27.72 ± 0.45	14.98 (14.94–15.03)	410.00	445.48 ± 16.59
20%	25.69 (25.65–25.74)	26.15 ± 1.29	15.31 (15.20–15.42)	393.38	424.67 ± 18.93
22%	24.48 (24.45–24.51)	25.10 ± 0.89	15.17 (15.09–15.26)	371.44	414.44 ± 10.42
24%	22.97 (22.96–22.99)	23.55 ± 0.58	16.05 (16.00–16.11)	368.75	410.00 ± 18.34
26%	19.61 (19.59–19.64)	20.23 ± 0.62	17.59 (17.49–17.70)	345.06	394.92 ± 12.49
28%	16.54 (16.51–16.56)	17.21 ± 0.36	17.85 (17.75–17.95)	295.22	350.41 ± 13.81
30%	15.20 (15.18–15.22)	15.87 ± 0.37	19.64 (19.52–19.77)	298.62	351.18 ± 22.58

## Data Availability

The original contributions presented in the study are included in the article, further inquiries can be directed to the corresponding author.
